# Vertebra segmentation based on two-step refinement

**DOI:** 10.1186/s40244-016-0018-0

**Published:** 2016-07-26

**Authors:** Jean-Baptiste Courbot, Edmond Rust, Emmanuel Monfrini, Christophe Collet

**Affiliations:** 1 ICube, Université de Strasbourg - CNRS, Illkirch, 67412 France; 2 SAMOVAR, Département CITI, CNRS, Évry, 91011 France

**Keywords:** Clinical imagery, Automatic vertebra segmentation, Coarse-to-fine modeling, SLIC clustering, Hidden Markov chain

## Abstract

Knowledge of vertebra location, shape, and orientation is crucial in many medical applications such as orthopedics or interventional procedures. Computed tomography (CT) offers a high contrast between bone and soft tissues, but automatic vertebra segmentation remains difficult. Hence, the wide range of shapes, aging, and degenerative joint disease alterations as well as the variety of pathological cases encountered in an aging population make automatic segmentation sometimes challenging. Besides, daily practice implies a need for affordable computation time.

This paper aims to present a new automated vertebra segmentation method (using a first bounding box for initialization) for CT 3D data which tackles these problems. This method is based on two consecutive steps. The first one is a new coarse-to-fine method efficiently reducing the data amount to obtain a coarse shape of the vertebra. The second step consists in a hidden Markov chain (HMC) segmentation using a specific volume transformation within a Bayesian framework. Our method does not introduce any prior on the expected shape of the vertebra within the bounding box and thus deals with the most frequent pathological cases encountered in daily practice.

We experiment this method on a set of standard lumbar, thoracic, and cervical vertebrae and on a public dataset, on pathological cases, and in a simple integration example. Quantitative and qualitative results show that our method is robust to changes in shapes and luminance and provides correct segmentation with respect to pathological cases.

## Background

Primitive bone tumors such as osteoid osteoma, metastatic lesions, and degenerative disorders such as arthritis or vertebral body collapse and traumatic injuries can affect one or several vertebrae. Diagnosis and characterization of these spine lesions rely on medical imaging. Computed tomography (CT) is yet one of the first-line imaging procedures. This cross-sectional imaging technique discriminates tissues along their densities and allows a good contrast between bones, surrounding organs, and soft tissues. However, identification of vertebrae can be difficult. Even if vertebrae vary in shape and orientation along the spine, these modifications can be slight between two neighbor elements of the backbone, making assessment of the exact level sometimes challenging.

A precise knowledge of vertebrae location, shape, and orientation is however essential. Hence, an imaging follow-up of spine lesions requires a precise identification of the affected levels and consequent reliable vertebrae identification. The same considerations are relevant in case of multi-modality imaging, that is to say supplementary spinal imaging procedures (e.g., bone scan with SPECT/CT, 18F-fluoride PET/CT) performed so as to allow a better characterization of lesions or tumor burden. This is even more crucial for preoperative planning and for interventional radiology treatments. Vertebra segmentation and identification is therefore a key issue for many medical applications.

Beside their ambiguous shapes and boundaries, one of the major concern in a segmentation perspective is the varying vertebrae neighborhood and shapes in a single patient, which led to the development of region-specific methods. Another important problem for clinical application is the eventuality of pathological cases, which is not always taken into account in previous works. This is challenging because of the wide range of diseases, e.g., on CT scans a spine lesion can affect the vertebra local shape (primitive tumor), global shape (scoliosis, fused vertebrae, degenerative disorders), or the intensity of some regions (hyper- or hypo-dense tumors). On top of that, one has to consider that a reliable vertebra segmentation method is one of the requirements needed to perform further advanced processing such as efficient image registration.

Medical image segmentation methods can be divided in three types: the iconic, the texture-based, and the edge-based methods [1]. Iconic methods rely directly on voxel intensities and include amplitude segmentation (e.g., thresholding) and region-based methods [2]. Texture-based methods rely on local operators [3] to describe and discriminate objects along their apparent texture. Edge-based methods use more abstract descriptors to constrain the shapes and boundaries. As mentioned in [4], vertebra segmentation is a challenging problem since vertebrae are inhomogeneous in intensity and texture and have complex shapes, which make traditional segmentation techniques inefficient to the problem. The vast majority of recent methods dedicated to vertebrae segmentation are edge-based and rely on deformable models performing an adaptation of prior data, such as templates or statistical atlases, to the vertebrae volume. For example, [5, 6] use a prior statistical shape model [7] as an initialization followed by a rigid or non-rigid registration, and in [4, 8, 9], the authors use a shape-constrained deformable model to fit a prior mesh into the data.

Nevertheless, two main key issues limit these works:

- The algorithms use complex shape description and processing, which dramatically increase global processing time.
- Methods are validated on a limited set of vertebrae in terms of scope (lumbar, thoracic, or cervical) and healthiness (middle-aged patient, healthy cases).

We assume in the following that vertebrae are properly isolated in bounding boxes, delimited roughly by their inter-vertebral disk and corresponding mean planes. Several methods of *vertebra localization* can produce such planes, such as the works presented in [4, 8, 10]. Since spine partition is not in the scope of this paper, the volume extractions are made manually. Therefore, this work focuses on the segmentation of vertebral elements contained in the volume, which may include parts of neighbor vertebrae.

Our method overcomes limitations listed above, since it does not rely on prior shape information or on complex shape descriptors. To restrain the computation time, we propose a coarse segmentation algorithm which drops voxel clusters from the data volume. This first step of the segmentation is built on the basis of a coherent voxel cluster statistical testing and is therefore robust to local and global luminance change. This coarse segmentation step will be referred as “Carving”. The second step aims at discriminating the two classes in the remaining volume within a robust Hidden Markov Chain (HMC) framework and thus performs coherent voxel-level segmentation. No shape priors are introduced in the algorithms; thus, the method can deal with any type of standard vertebrae from lumbar to cervical as well as with most of the non-standard cases one can expect in clinical context. In the Sections “Coarse segmentation” and “Fine segmentation based on HMC modeling” the two-pass segmentation algorithm is described. The Section “Results” explains the experiments and the results obtained with the proposed method. A discussion on the results is given in the Section “Discussion and conclusion” as well as a conclusion on the method.

## Method

### Coarse segmentation

**Fig. 1:**
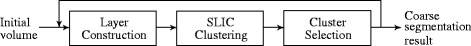
Flowchart of the coarse segmentation algorithm
- The *layer clustering* produces clustered voxels with a joint space-luminance criterion.
- The *cluster selection* tests if the clusters should be rejected or included in the final volume.

In the context of medical image processing, coarse-to-fine methods are mostly used to perform fast registration (see, e.g., [11] or [9]) as they reduce computation time. On the other hand, image clustering is a well-known tool grouping individual elements (*i*.*e*., voxels) following a specific similarity criterion [12] (based on a given distance metric) and thus produces consistent high-level elements. In our case, it is desirable to combine both approaches to rapidly ensure a first accurate and consistent estimation of the anatomical vertebral volume. Therefore, a new algorithm is introduced to perform a coarse segmentation of the data volume within a previously delimited bounding box [4, 8, 10]. It processes the volume layer by layer iteratively, following three steps (see Fig. 1):

- The *layer construction* consists in selecting the external layer to be processed from the volume of interest in the current iteration.

The three steps are repeated until the volume is completely processed within the initial bounding box.

#### Layer construction

This step isolates the external layer of voxels on which the further processing will be applied. The first layer is defined by its depth *I*_1_ from the borders of the volume. Given the boundary from the previous step, the following layers cover both an inner part of depth *I*_*j*_ and an outer part of height *O*_*j*_, *j*>1. The layer are isolated with mathematical morphology operator.

More precisely, let $\hat {V}_{j-1}$ be the binary partially segmented volume obtained at the step (*j*−1) and *R*(*a*) be a ball structuring element of radius *a*. The layer *V*_*j*_ at the *j*-th iteration is then defined as:

1 $$ \begin{aligned} V_{1} &= V_{0} - V_{0} \ominus R(I_{1}) \\ V_{j} &= V_{j-1} \oplus R(O_{j}) - V_{j-1} \ominus R(I_{j}) ~\forall j>1 \end{aligned}  $$

**Fig. 2:**
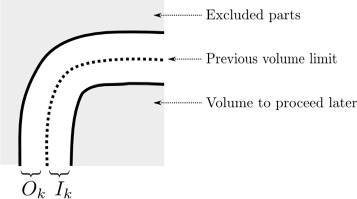
2D illustration for layer construction at the *k*-th iteration. *Shaded parts* are not processed at this step and are already excluded (*outer part*) or not proceeded yet (*inner part*)

*V*_0_ is the initial bounding box, and the operators ⊕ and ⊖ stand for morphological dilatation and erosion, respectively^1^. The external layer is used to avoid artifacts creation by processing the layer boundary regions at least twice. Figure 2 illustrates the layer construction step.

#### Layer clustering

We develop a clustering method based on the simple linear iterative clustering (SLIC) method proposed by Achanta et al. [13]. The authors presented a clustering method for color images we generalize in the 3D gray level case. Thereafter, it will be referred as “SLIC-3D”. In the color image case, a pixel *i* can be defined by its Cartesian coordinates (*x*_*i*_, *y*_*i*_) and the *L*∗*a*∗*b* intensities (*l*_*i*_,*a*_*i*_,*b*_*i*_). In [13], the authors combine the two representation spaces in one distance using two weighting parameters called *m* and *S*: *m* is used to balance the contributions of the color distance with respect to the Euclidean distance and *S* stands for the number of pixels a super-pixel is expected to contain. The SLIC algorithm proposed in [13] consists in clustering the pixels in order to approximately minimize for each pixel its combined distance to the cluster centroid.

We propose on a similar principle a clustering algorithm addressing 3D data. The Euclidean distance covers then a 3D space and the luminance of a voxel stands for the color channel. As CT data is processed, its intensity is expressed in Hounsfield Units (HU) corresponding to X-ray absorption ratio of organic tissues with respect to water [14]. A given voxel *i* is then represented by its spatial coordinates (*x*_*i*_,*y*_*i*_,*z*_*i*_) and its luminance *l*_*i*_. For a given voxel cluster *k* containing |*k*| elements, we define its centroid *C*_*k*_ as its mean value along the four features:

2 $$ C_{k} = [x_{k}, y_{k}, z_{k}, l_{k}]^{T} = \frac{1}{|k|}\sum_{i \in k}[x_{i}, y_{i}, z_{i}, l_{i}]^{T}  $$

Then, a mixed distance *D*_*m*_ combining the four features between a cluster centroid *C*_*k*_ and a voxel *i* is given by:

3 $$ D_{m}(C_{k},i) = \sqrt{\left(\frac{d_{c}(C_{k},i)}{m}\right)^{2} + \left(\frac{d_{s}(C_{k},i)}{S}\right)^{2}}   $$

where *d*_*s*_ is the 3D Euclidean distance and *d*_*c*_ is a luminance distance between *C*_*k*_ and *i*:

4 $$ \begin{aligned} d_{s}(C_{k},i) &= \sqrt{(x_{k} - x_{i})^{2} + (y_{k} - y_{i})^{2} + (z_{k} - z_{i})^{2}} \\ d_{c}(C_{k},i) &= |l_{k} - l_{i}| \end{aligned}  $$

The cluster centroids are initialized on a regular cubic grid of size *S*. For each cluster, the algorithm processes a cubic 2*S*×2*S*×2*S* region centered on the centroid spatial coordinates. Each voxel in the region closer to the cluster centroid than to its current cluster centroid is then re-labeled. Finally, the cluster centroids are updated, and the procedure can be repeated for a few iterations. The SLIC-3D procedure is summarized in Algorithm 1.



#### Cluster selection

The clusters from a given layer need to be tested to assess if they belong to the vertebra volume. Since vertebrae are mainly bone tissue and have typical luminance in CT volume, the test is built with the mean luminance *l*_*k*_ of each cluster *k*. Thus, voxels are accepted or rejected by cluster, avoiding to deal with local irrelevant voxel variations. Furthermore, the test must be robust to changes in the vertebrae to proceed. Thus, simple test such as thresholding are not satisfying and a more elaborate procedure is needed. This is why an adaptive test is developed to ensure robust and consistent clusters acceptance or rejection. The test is based on the *statistical region merging* approach, proposed by Nock and Nielsen [15]. Whereas the authors use the test for a pixel pair set, we propose to test the clusters with respect to a *reference luminancel*_0_ corresponding to the typical bone luminance in CT scans. For a given cluster *k*, let a first *bone merging* predicate be:

5 $$  \mathcal{P}_{0}(k): |l_{k} - l_{0} | \leq b(k)  $$

where *b*(·) is a *merging threshold* [15]:

6 $$ b(k) = g \sqrt{\frac{1}{Q|k|} ln \left(\frac{1}{\delta} \right)}  $$

where |*k*| is the number of voxels in the cluster *k*, *g* is the grey level range, and *δ* is the acceptable probability of error for the predicate. *Q* stands for the expected number of underlying independent random variables (r.v.) for the current region, and according to [15], it allows to quantify its *statistical complexity*. The *bone merging* predicate can then be stated as “*accept the cluster k if* |*l*_*k*_−*l*_0_| < *b*(*k*)”.

Furthermore, an alternative is added to the test: vertebrae being compact objects, each *interior* cluster is necessarily part of the result. A predicate is needed to assert for a given cluster, the *interiority* of its neighbors. We define as interior to the remaining volume any point closer to the remaining volume center *v*=(*x*_*v*_,*y*_*v*_,*z*_*v*_) than the centroid of the supervoxel. Then, an *interiority* predicate is built for a given cluster *k* and any of its neighbor *k*^′^:

7 $$  \mathcal{P}_{I}^{k}(k'): d_{s}(C_{k'},v) \leq d_{s}(C_{k}, v)  $$

where *C*_*k*_ and $C_{k^{\prime }}\phantom {\dot {i}\!}$ are the centroids of the clusters *k* and *k*^′^, respectively, and *d*_*s*_ is the Euclidean distance defined in (4). Finally, the two predicates (5) and (7) are combined in the following *vertebral* predicate to test a cluster *k*:

8 $$ \mathcal{P}(k): \mathcal{P}_{0}(k) \text{~or~} \mathcal{P}_{I}^{k'}(k) ~ \forall k' \text{~such as} \left\{\begin{array}{l} k \text{~and~} k' \text{are neighbor,} \\ \mathcal{P}(k') \text{~is valid.} \end{array}\right.  $$

**Fig. 3:**
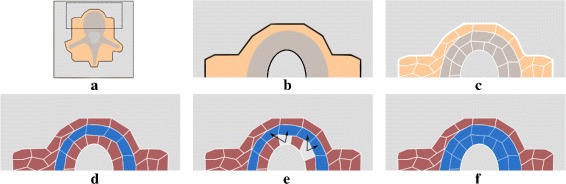
Graphical summary of a coarse segmentation step. *Gray regions* are not proceeded at this step, *orange regions* are the current layer and include the *brown* vertebrae region. White limits represent cluster boundaries. **a** Whole slice with the zoom-in region **b** in the dotted limits. **c** Result from the SLIC-3D clustering. **d** Possible outcome of the selection step with only the bone merging predicate (5). *Blue* accepted clusters, *red* rejected clusters. **e** Neighborhood search example for the two *light-gray* clusters **f** Expected outcome of the vertebral predicate (8)

This predicate allows an efficient selection of voxel cluster. Figure 3 illustrates the whole coarse segmentation step.

#### Model and parameters

The model requires calibrating several parameters. They were evaluated on a first set of 12 lumbar thoracic and cervical vertebrae. Motivations are given below:

- Equation (1) defining the layer construction and the SLIC-3D method (Algorithm 1) both depend on a size parameter. We choose to define them only with the *S* parameter from SLIC-3D, meaning that we link the layer depth to the expected size of supervoxels. Thus, this parameter quantifies both the depth of the current layer and the scale of the supervoxel to exclude. We choose the two first values of *S* to be higher than the latter ones, as we process in a coarse-to-fine fashion. *S* is given in millimeters to ensure isotropy between axes and between scans.
- The *m* parameter used in clustering (Algorithm 1) mostly defines the clusters shape between a spatial regularity and an intensity regularity. We use decreasing values of *m* along iterations to exclude first spatially coherent and then intensity-coherent clusters.
- The statistical parameters *g*, *Q*, and *δ* used for cluster selection (Predicate 5) can be computed automatically on the basis of the cluster to proceed: *g* is the range of the current layer intensity and *Q* must be set lower than *g* to reduce the expected complexity of the bone merging predicate. The error probability *δ* can be fixed arbitrarily, e.g., as the inverse of the cardinal of the cluster.
- The reference intensity *l*_0_ (Predicate 5) is the intensity of typical bone in CT scans and is provided by an expert.

**Fig. 4:**
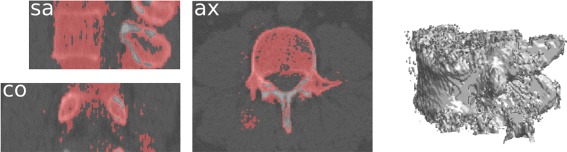
Result of the coarse segmentation step for a L3 vertebra. The three sectional views are the sagittal, axial, and coronal middle slice of the source volume. The result is represented by its red superimposition and the 3D interpolation of the coarse segmentation volume



The algorithm is built on the three steps previously detailed and processes the volume iteratively. For a volume of height *h*, the number of iterations *J* is given by^2^:

9 $$ J = 2 + \left\lceil \frac{h - S_{1} - S_{2}}{S_{j}} \right\rceil   $$

where ⌈·⌉ is the ceiling operator, *S*_1_, *S*_2_, and *S*_*j*_ stands respectively for the two first values of *S* and its value for any iteration *j*>2. The model is tolerant to parameter variations, as long as their order from coarse to fine is preserved. Note that variations of the *S* parameters may produce changes in terms of computation time, since it influences the number of iteration to proceed in (9). In all cases, we observed convergence towards similar results. The entire coarse segmentation method that we called Carving, as well as the parameters value, are summarized in Algorithm 2. Figure 4 illustrates the results obtained at this step.

The result of the coarse segmentation is very nice given the expectation: the first need is to reduce the data amount to proceed, which is efficiently done. The algorithm actually does more than data volume reduction since the results already have the shape of the underlying vertebrae. However, this step alone remains too coarse, and we have now to use a finer segmentation to perform a voxel-level classification of the remaining volume.

### Fine segmentation based on HMC modeling

The coarse segmentation results obtained at the previous section are smaller than the initial volume and include most of the anatomical vertebrae volume. However, to allow an efficient final segmentation, we need to have in the volume enough voxels of the two classes to separate. Thus, a region of interest (ROI) is built based on the previous coarse result. It is a morphological dilation with a ball structuring element of radius 10 mm. In this section, this ROI will be processed, as it preserves the expected shape of the vertebra and includes enough non-vertebral voxels to allow automatic separation.

We are interested in a robust, voxel-wise segmentation method. The Bayesian framework meets these requirements and offers a consistent statistical modeling for the segmentation of an image into classes. When processing images or volumes, Hidden Markov Random Field (HMRF) [16] modeling often provides good results because the model do consider spatial relationships. However, using HMRF can be computationally time-consuming. This is mainly due to the sampling needed to perform estimations from an analytically unknown distribution. On the other hand, the classical HMC framework, while having the advantages of Bayesian segmentation, does not have the drawbacks of HMRF. It provides faster computations, and we use it with a specific volume transformation to preserve the most important spatial features. The Baum-Welch algorithm [17] is used for segmentation, based on parameters estimated with the Stochastic Expectation-Maximization (SEM) [18] method.

#### Volume transformation

First of all, the 3D data needs to be transformed to obtain a one-dimensional chain. This point must be carefully considered, since while it permits fast computation, it introduces an artificial 1D order in 3D data and thus uses only 2 out of 26 neighbors for each voxel. A 3D volume can be transformed by sweeping each line, column, and row from first to last but this transformation induces too much distortion to the original data structure. Another alternative is the Hilbert curve [19], which is known to be successful for transforming 2D or 3D images into chains (see, e.g., [20–22]). The resulting chain is more spatially regular; however, it creates artifacts in the HMC segmentation because the chain requires having relatively few state transitions to produce a smooth segmentation estimate. The Hilbert curve path does not ensure this; therefore, in this section, a new volume-to-chain transformation is introduced, relying on the shape information obtained at the coarse segmentation step.

**Fig. 5:**
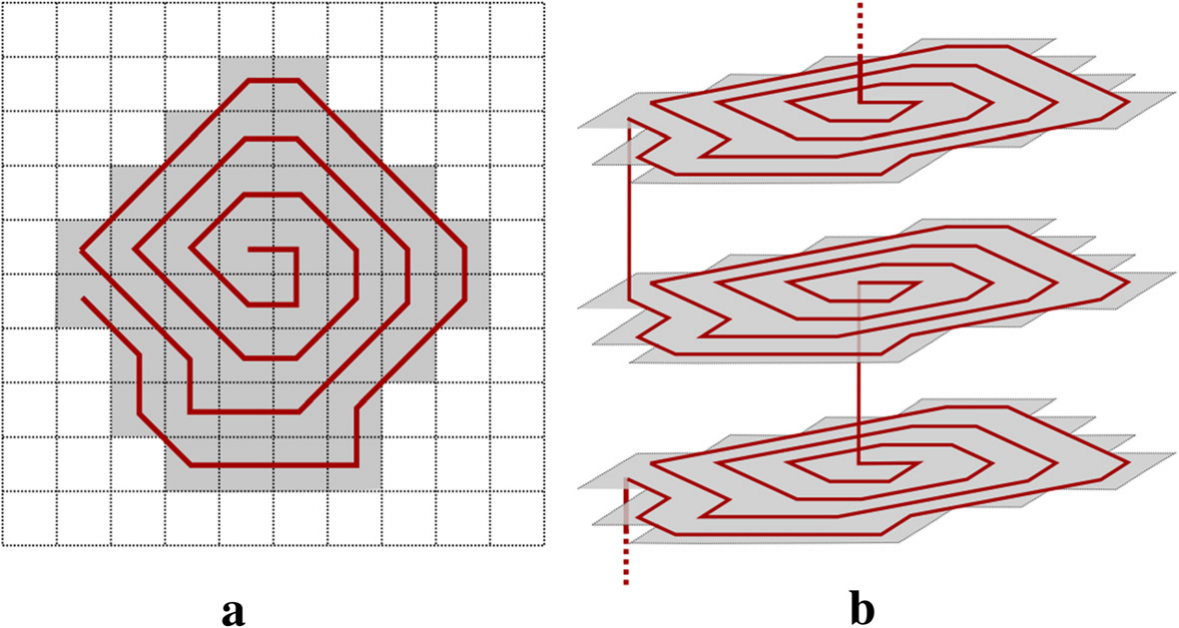
Spiral transform illustration. The *shaded regions* represent the ROI sections, and the *red line* follows the chain path. **a** Example of a 10×10 pixel slice. **b** Example for three consecutive slices



The volume is processed by slices. Given the symmetries of a vertebra, horizontal slices are retained: the axis of our spirals is axial^3^. For each slice, a spiral along concentric perimeters of the ROI section is built. The spiral path goes alternatively inward and outward, so that consecutive spiral extremities are spatially close from one slice to another. Algorithm 3 summarizes the process, which is illustrated in Fig. 5.

#### Forward-backward algorithm

This algorithm allows the computation of posterior densities required for segmentation. Let *N* be the length of the chain obtained from the spiral transform. ***X***=(*X*_1_,…,*X*_*N*_) and ***Y***=(*Y*_1_,…,*Y*_*N*_) are respectively the random variables sequences representing the spiral transformation of the class volume and the observed volume. We will note ***x***=(*x*_1_,…,*x*_*N*_) and ***y***=(*y*_1_,…,*y*_*N*_) their respective realizations. The class volume elements take their values in *Ω*={*ω*_0_,*ω*_1_} since we want to discriminate vertebral (*ω*_1_) from non-vertebral (*ω*_0_) elements. The observed volume voxels remain in Hounsfield Units, typically in a range of [ −2000, 2000] HU. For clarity, we note *p*(*x*_*n*_) and *p*(***x***) instead of *p*(*X*_*n*_=*x*_*n*_) and *p*(***X***=***x***), respectively, and likewise for the ***Y*** process.

We assume that (***X***,***Y***) is a HMC with independent noise (HMC-IN). The following properties are verified:

- ***X*** is a Markov chain:
  10 $$ p(\boldsymbol{x}) = p(x_{1}) p(x_{2}\,|\,x_{1}) \ldots p(x_{N}\,|\,x_{N-1})   $$
- The (*Y*_*n*_)_1≤*n*≤*N*_ are conditionally independent with respect to ***X***:
  11 $$ p(\boldsymbol{y}\,|\,\boldsymbol{x}) = \prod_{n=1}^{N} p(y_{n}\,|\,\boldsymbol{x})   $$
- The noise independence is verified :
  12 $$ p(y_{n}\,|\,\boldsymbol{x}) = p(y_{n}\,|\,x_{n}) ~ \forall n \in \left\{ 1, \ldots, N \right\}  $$
- The previous points lead to the following expression for the joint (***X***,***Y***) probability distribution:
  13 $$ p(\boldsymbol{x}, \boldsymbol{y}) = p(x_{1}) p(y_{1}\,|\,x_{1}) \prod_{n=2}^{N}p(x_{n}\,|\,x_{n-1}) p(y_{n}\,|\,x_{n})   $$

Then the classical *forward-backward* decomposition [17] of the posterior marginal probability yields:

14 $$ \xi(x_{n}) = p(x_{n}\,|\,\boldsymbol{y}) = \frac{\alpha(x_{n}) \beta(x_{n})}{\sum\limits_{\omega \in \Omega} \alpha(\omega) \beta(\omega)} ~ \forall n \in \left\{ 1, \ldots, N \right\}   $$

Where *α* and *β* are respectively the *forward* and *backward* probabilities for *n*∈{1,…,*N*}:

15 $$ \begin{aligned} \alpha(x_{n}) &= p(x_{n}, y_{1}, \ldots, y_{n}) \\ \beta(x_{n}) &= p(y_{n+1}, \ldots, y_{N} \,|\,x_{n}) \end{aligned}  $$

Both *α* and *β* can be computed using the following recursions:

16 $$ \begin{aligned} \text{\textit{Initializations}:} &\left\{ \begin{array}{ll} \alpha(x_{1}) = p(x_{1}, y_{1})\\ \beta(x_{N}) = 1 \end{array}\right.\\ \text{\textit{Inductions}: }\\ \forall 1 \leq n \leq N-1 : &\left\{\begin{array}{ll} \alpha(x_{n+1}) = \left(\sum\limits_{\omega \in \Omega} \alpha(\omega) p(x_{n+1} \,|\, \omega) \right) p(y_{n+1}\,|\,x_{n+1})\\ \beta(x_{n}) = \sum\limits_{\omega\in \Omega} \beta(\omega) p(\omega \,|\, x_{n}) p(y_{n+1}\,|\,\omega) \end{array}\right.\\ \end{aligned}  $$

The posterior marginal probabilities (14) can then be computed, and the maximum posterior mode (MPM) class estimation [23] yields:

17 $$ \forall 1 \leq n \leq N ~~\hat{x}_{n}= \textit{{arg}} ~ \underset{\omega \in \Omega}{max}~ p(X_{n} = \omega \,|\, \boldsymbol{y})   $$

The distribution in (16) requires the knowledge of noise and model parameters. In an unsupervized segmentation framework, they must be estimated. An estimation method is reported in the next section.

#### Parameter estimation

The parameters from Eq. (13) need to be estimated to perform the class estimation. We note:

18 $$ \begin{aligned} p(X_{1} = \omega_{i}) &= \pi_{i} \\ p(X_{n} = \omega_{j} \,|\, X_{n-1} = \omega_{i}) &= \pi_{ij} ~ \forall n \in \left\{ 1, \ldots, N \right\} \end{aligned}  $$

Note that the *π* parameters do not depend of *n* since we supposed the HMC to be homogeneous. We use the SEM algorithm [18] to perform the parameter estimation. SEM is chosen over its determinist counterpart EM [24] for robustness reasons, since the algorithm needs to efficiently deal with non-standard cases (e.g., noise, pathologies, artifacts) and to avoid local extrema convergence. We assume that we are in the case of a Gaussian mixture:

19 $$ p(y_{n}\,|\,X_{n}=\omega_{i}) \sim \mathcal{N}(\mu_{i}, \sigma_{i})\text{~with }i \in \left\{ 0, 1 \right\} \text{for~} n \in \left\{ 1, \ldots, N \right\}  $$

The set of parameter to estimate is ***Θ***={*π*_*ij*_,*π*_*i*_,*μ*_*i*_,*σ*_*i*_} for *i*,*j*∈{0,1}. With complete data (***x***,***y***), one can perform the estimation of ***Θ*** with the maximum likelihood (ML) estimators. Complete data are however unavailable. This is why SEM iteratively provides simulations of ***x*** along posterior distributions.

The SEM algorithm requires an accurate initialization to ensure fast parameter estimation convergence. At this step, we split the process: in some known cases, the volume can include air elements, which are clearly distinct from both soft tissues and bones. To avoid wrong class clustering, we use for the initialization a set of reference parameters obtained from other vertebra of the same patient without air in the neighborhood. This allows correct calibration with respect to the patient and the scanner, while avoiding wrong class clustering. In any other cases, we use a simple initialization where *μ*_0_=0.25, *μ*_1_=0.75, and *σ*_0_=*σ*_1_ are estimated through the ML estimator from the whole sequence **y** and *π*_*ij*_=*π*_*i*_=0.5 ∀*i*,*j*∈{0,1}. These initializations are choosen to ensure class separation and avoid reliance on other algorithm(s) convergence (e.g., the K-means algorithm [25]).

For the simulation (S) step, one needs to compute the posterior transition probabilities, given by:

20 $$ \begin{aligned} p_{\boldsymbol{\Theta}^{k}}(\boldsymbol{x} \,|\, \boldsymbol{y}) &= p(x_{1} \,|\, \boldsymbol{y}) \prod_{n=2}^{N} p(x_{n}\,|\,x_{n-1}, \boldsymbol{y}) \\ p(x_{n}\,|\,x_{n-1}, \boldsymbol{y}) &= \frac{p(x_{n} \,|\, x_{n-1})p(y_{n}\,|\,x_{n}) \beta(x_{n})}{\sum\limits_{\omega \in \Omega} p(\omega \,|\, x_{n-1})p(y_{n}\,|\,\omega) \beta(\omega)} ~\forall 2 \leq n \leq N \end{aligned}  $$

An additional step in the original SEM procedure is introduced to produce a convergence measure for the parameters estimate. Since the individual *π*, *μ*, and *σ* parameters differ in nature, we cannot use a direct Euclidean distance comparison between two consecutive estimations of ***Θ***. However, the forward-backward class estimation provides for a given ***Θ*** and fixed observations ***y*** a determinist result. The convergence between two SEM steps is then estimated through the variations between consecutive on-the-fly forward-backward estimates based on the consecutive parameter estimation. The convergence rate between two consecutive parameter estimations ***Θ***^*k*^ and ***Θ***^*k*−1^ is computed together with their corresponding forward-backward estimates $\hat {\boldsymbol {x}}^{k}$ and $\hat {\boldsymbol {x}}^{k-1}$, respectively, as:

21 $$ \epsilon = \frac{1}{N} \sum_{n=1}^{N} \left(\hat{x}_{n}^{k} - \hat{x}_{n}^{k-1}\right).  $$

We assume that the SEM algorithm has converged when *ε*<1 *%*. This choice allows performing the algorithm in a small number of iterations: typically less than 15 iterations are needed. Setting a smaller *ε* increases the global processing time and does not provide noticeable improvement to the result. The adapted SEM algorithm is summarized in Algorithm 4.



#### HMC segmentation algorithm

**Fig. 6:**
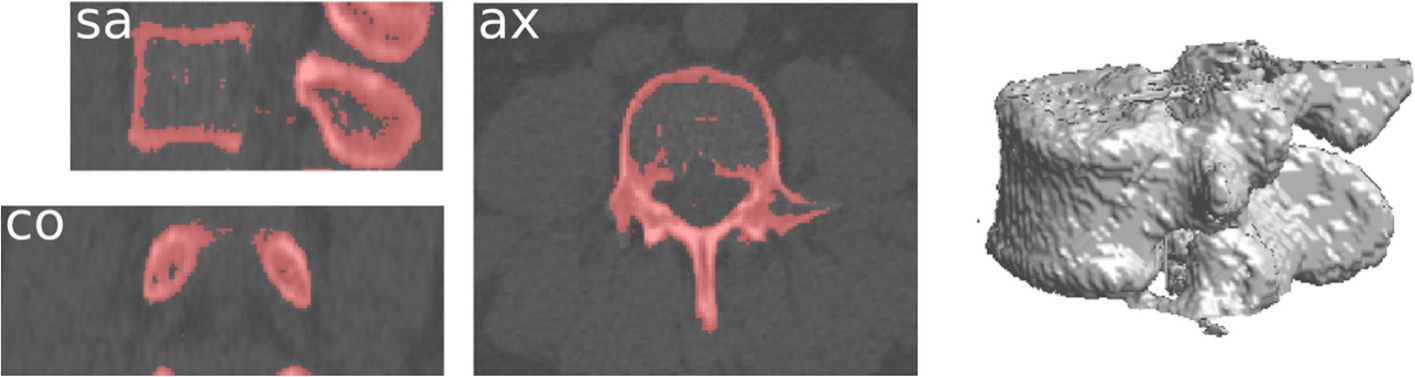
Result of the segmentation step for a L3 vertebra. It follows the coarse result presented in Fig. 4, with the same legend



First, the segmentation algorithm transforms the volume along a spiral path adapted to the coarse shape. The parameters of the mixture are then estimated with the SEM algorithm. Once the mixture estimation is done, posterior marginal probabilities (14) can be computed to find the MPM estimation (17) of the observed vertebra. Finally, the segmented volume is re-built along the initial chain path. Algorithm 5 summarizes the segmentation procedure, and Fig. 6 illustrates the result for the segmentation following the coarse result from Fig. 4.

**Fig. 7:**
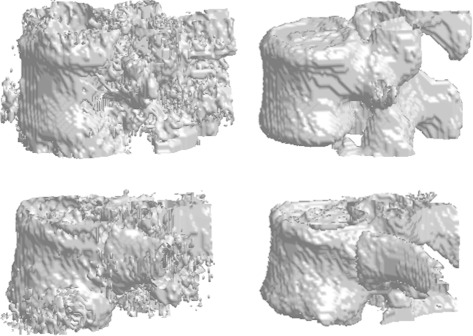
3D interpolations of the coarse and fine segmentation results. The first and second rows correspond to lumbar (L4) and thoracic (T11) vertebrae processing, respectively. The first column represents the result of the Carving method (Algorithm 2), and the second column contains the results after the HMC segmentation (Algorithm 5). Note that the observed granularity corresponds to the voxel size, which is the minimal size addressed in this work

Complementary results are presented in Fig. 7. The gain of the HMC segmentation step is clear from the 3D interpolations: the results match our expectations of the vertebral volumes and does not include processing artifacts. Note that while the HMC excludes the inner part of the vertebral body for some lumbar vertebrae (e.g., the vertebra from Fig. 6), the border boundaries are well separated. This is not a key topic for a localization purpose, but other goals (e.g., biomechanical modelling) may require some post-processing in these cases. Further extensive and comparative results are presented in the next section, as well as robustness examples and whole-spine segmentations.

## Results

In this section, the method performance is evaluated. First, the method is qualitatively evaluated on a set of 339 standard vertebrae acquired in daily practice. Then, quantitative results on manually segmented data are reported. Pathological cases make then the robustness evaluation possible. Finally, we provide simple integration examples with a segmentation of the full spine.

### Standard cases: qualitative results

The method is evaluated on a set of vertebral volumes from the whole spine of 15 consecutive patients in an oncologic tertiary center, with exclusion of patients with bone tumors or metastatic spine involvement. Patients had a mean age of 63 and presented degenerative joint alterations and some osteoporotic changes, reflecting most of the situations encountered in daily practice; 339 vertebral volumes were extracted and evaluated, meaning that almost all patients’ vertebrae were tested.

Each volume is applied successively Algorithm 2 for coarse segmentation and Algorithm 5 for fine segmentation. For the sake of comparison, the K-means algorithm is used as a benchmark since it has common ground with the proposed method: it processes the voxel intensities and has no prior on the volume shape. Since the two-class K-means classification fails when air is present in the volume, we use a sub-sample in which no air is present to provide a more accurate comparison.

For this data, there is no available ground truth (e.g., manual segmentation). Therefore, one must resort to qualitative evaluation. We define, in a similar fashion than in [4], the following ranking:

- *Excellent* (100): the vertebra is exactly delimited inside its bounding box.
- *Good* (75): most of the anatomical structure is covered, but some voxels are segmented out.
- *Bad* (50): the vertebra is recognizable but noticeable part are missing from the result.
- *Poor* (25): the vertebra is not recognizable enough.
- *Fail* (0): the segmentation fails to proceed.

The results were visually inspected by an expert with respect to these criteria. Table 1 summarizes the results obtained for the proposed method and the K-means method. Considering that both good and excellent results provide sufficient data for vertebrae segmentation and further advanced processing, our method provides about 78 % of successful results on the subsample where K-means gives 72 % of successful results, whereas on the full sample, the method yields 67 % of successful result and the 2-class K-means fails on the remaining 161 volumes including air (yielding thus an average score of 37.76 % on the full sample). Keeping in mind that the sample originates from daily routines and includes a significant proportion of minor pathologies, these results are of significant interest for clinical use.

**Table 1:** Results for the subsample without air and for the full sample. This parting provides accurate comparative results on the sub-sample

	Partial set: 178 volumes		Full set: 339 volumes
Grade (score)	Proposed method	K-means	Proposed method
Excellent (100)	75 (42.13 %)	46 (25.84 %)	98 (28.91 %)
Good (75)	64 (35.96 %)	83 (46.63 %)	129 (38.05 %)
Bad (50)	31 (17.42 %)	35 (19.66 %)	80 (23.60 %)
Poor (25)	6 (3.37 %)	9 (5.06 %)	29 (8.55 %)
Fail (0)	2 (1.12 %)	5 (2.81 %)	3 (0.88 %)
**Average score**	**78.65**	**71.91**	**71.39**

The algorithms were developed and tested using Matlab on an Intel i5 (2.6 GHz) on one core, without specific optimization of the code. The processing time is of 36 s by vertebra on average, with 10 s for the Carving step and 26 s for the HMC step. This processing time depends on the size of the vertebra to segment, the average total time being 71.4 s for lumbar vertebrae and 19.8 s for cervical vertebrae. For daily practice implementation, the use of C/C++ language is expected to provide a gain of a factor at least 10 to the processing time.

**Fig. 8:**
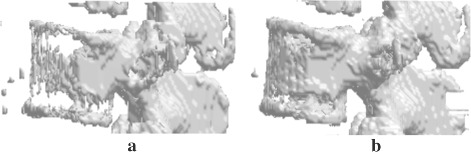
Illustrative comparison between K-means segmentation (**a**) and the proposed method (**b**) for the L3 vertebra in a patient with marked osteoporosis. K-means segmentation is rated 50, whereas the proposed segmentation is rated 75

Our method yields satisfying results. Noteworthy, bad and poor results provided by the method are mainly encountered in levels with either pronounced degenerative joint diseases alterations or marked osteoporosis with consequent low contrast (see Fig. 8). In such cases, our method provides better results than the K-means segmentation, supporting the evidence that our proposal is more reliable in data reflecting daily practice. In only 6.5 % of the cases, the K-means segmentation provided better results than our method.

The results presented here cover standard volumes mostly encountered in practice, but did not yield voxel-wise error rate. The next section presents a quantitative evaluation of the method, with respect to manual segmentations.

### Quantitative results

When considering vertebral segmentation in CT images, there are few available complete dataset allowing a quantitative evaluation of a method. We use the dataset presented at the CSI 2014 challenge [26], available on the public SpineWeb platform [27]. This dataset contains 10 spine scans from a trauma center, acquired during daily clinical routine work. Patients were aged 16 to 35, and the scans covered thoracic and lumbar vertebrae in most cases. A total of 175 segmented volumes were extracted from the dataset. The performances are measured as the rate of correctly classified voxels (true positives and true negatives).

**Fig. 9:**
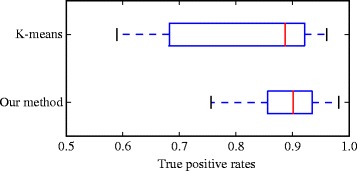
Box plots for the quantitative results. The average rates are 0.816 for the K-means segmentation and 0.894 for the proposed method (see text)

**Fig. 10:**
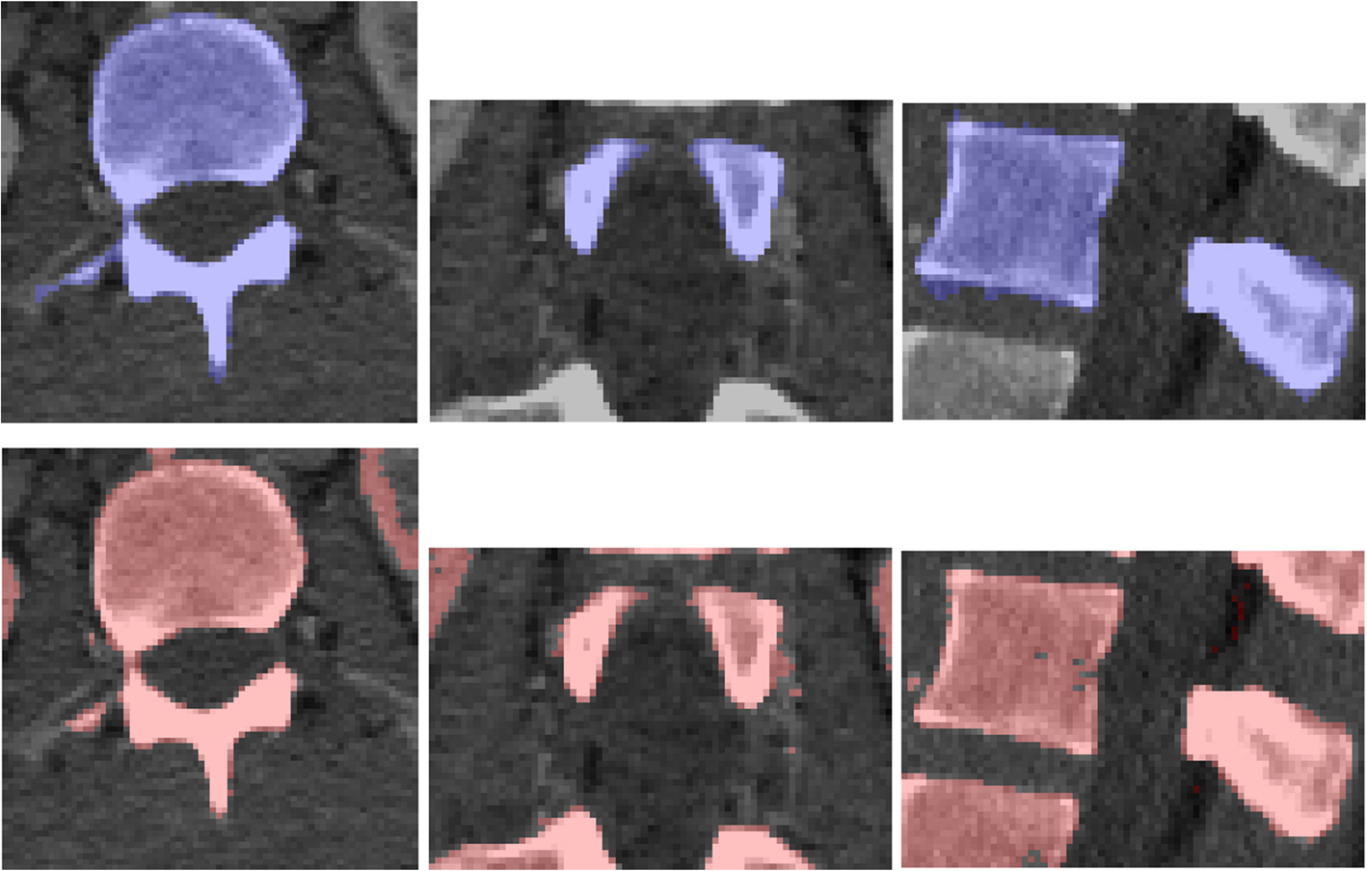
Ground truth (*first row*) and segmentation (*second row*) on a L1 vertebra. Source volume and colored superimposed results are displayed from left to right within the median axial, coronal, and sagittal planes. In this case, the correct segmentation rate is 94.19 *%*

Figure 9 summarizes the results with respect to the provided vertebra segmentation. On average, the proposed method yields 89.39±5.54 *%* of correct segmentation. On the same dataset, the K-means segmentation provides 81.65±12.23 *%* correct segmentation. These results imply that our method provide both good results and a small variability on the output. Note that these results concern only the vertebra of interest: a correctly labeled neighboring vertebra is ignored in the measurements. An illustration of the ground truth compared to a segmentation result is provided in Fig. 10.

Errors are either false positive (type I error) or false negative (type II). False positive are most encountered in the presence of high-intensity elements, such as calcifications or ribs near thoracic vertebrae. On the other hand, false negatives are either missing voxels at the vertebrae boundary or missing voxels within the vertebral body.

These results are satisfying, given that most error sources are known and that specific post-processing could easily remove them. So far, the performances were evaluated on standard cases: the next section presents pathological cases that may be encountered in practice.

### Pathological cases: robustness evaluation

As our method performs correctly on standard case, it has to be evaluated in more difficult situations, namely pathological cases. Since we aim at a clinical implementation, the method robustness to the most frequent non-standard cases is indeed mandatory. We first briefly describe the selected cases and the corresponding challenges, then we provide their corresponding segmentation results and discussion.

**Fig. 11:**
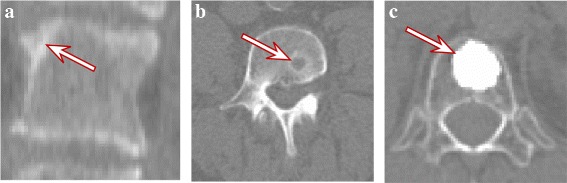
**a** coronal and **b**, **c** axial sectional views of the selected cases. The *arrows* highlight their specificities (see text for details)

The two main key points are changes in shape and in intensity of the object to segment. They correspond to anatomical and structural deformations, respectively. Structural changes are related to alterations of bone and medullar matrix, with consequent modification of density and signal intensity in the CT volume. Changes of shape and density can be related to aging alterations. In particular, arthrosis is responsible of spine alterations in a general population, and we selected it as the first specific case (see Fig. 11a). The frequency and intensity of these modifications is in close relationship with age. After 40 years, hernia, osteophytes, and degenerative joint diseases are commonly encountered. We also selected a hernia case as an instance of common low-intensity structural alteration (see Fig. 11b).

On the other hand, many pathologic conditions can lead to bone density variations. For instance, osteoblastic cancerous tumors will increase bone density. On the other side, osteolytic tumoral involvement is associated with bone destruction and is therefore seen as areas of decreased bone density. Finally, treatments—general treatments as chemotherapy or interventional treatments as cementoplasy—can induce bone density alterations. In particular, cementoplasty, which can be described as the interventional introduction of artificial high-density material inside the vertebral body, represents an extreme case of overdensity. This is the third case we retained (see Fig. 11c).

**Fig. 12:**
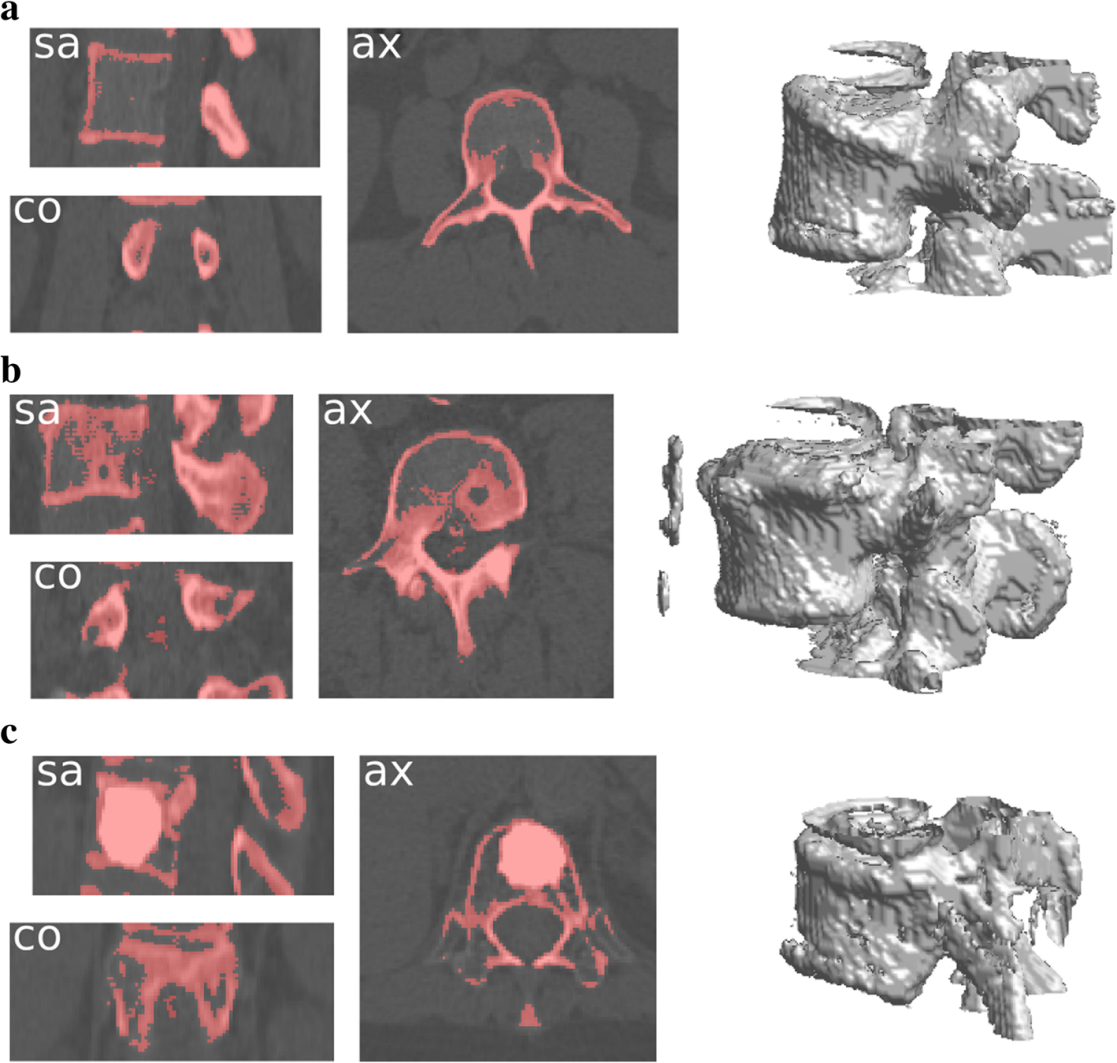
Results of the proposed segmentation method on the selected particular cases. They include parts of the upper and lower vertebrae as they appear in the bounding boxes. **a** Arthrosis on a L3 vertebra. Boundaries differs in width and in shape from a typical lumbar vertebrae. **b** Hernia in a L4 vertebra. Note that some calcifications are also segmented since their intensity is close to the bone intensity, and they are located near the vertebral body. **c** Cementoplasty in a T12 vertebra
- The hernia case presents a region which can be seen as a vacuum in the bone material. This vacuum is segmented out by the method since it differs from the bone in intensity. However, this particular point does not prevent the method to perform the segmentation correctly. Noteworthy, some inner parts of the vertebral body are included in the segmentation, which is not the case for standard lumbar vertebrae (e.g., Figures 6,12a). This is due to the relative overdensity induced by the hernia in the surrounding material.
- Finally, the cementoplasty case represents a more challenging test. It produces indeed an almost homogeneous bright region inside the vertebral body, leading to a global distortion of the data in comparison with the standard cases. Nevertheless, the proposed method successes in providing a correct result (Fig. 12c), which does not cover the full vertebra volume but does represent most of the underlying vertebra. It also shows that natural overdensities of lower range can be handled by our method, the cementoplasty being one of the most extreme cases.

Figure 12 provides the results on the three selected cases. They are discussed below:

- From Fig. 12a, it appears clearly that the changes of the vertebral body boundaries do not impede the segmentation result, which is similar to the result presented in Fig. 6. Some imprecisions appears in the 3D interpolation; however, they are minor given the overall result.

The results presented here show that the proposed method is robust to some of the most frequent particular cases met in clinical context. Furthermore, as it provides a correct result for a challenging case, one can expect it to be robust to most of the lower-intensity specificities. We provide in the next section further results covering the range of all vertebrae for two patients.

### Integration examples

**Fig. 13:**
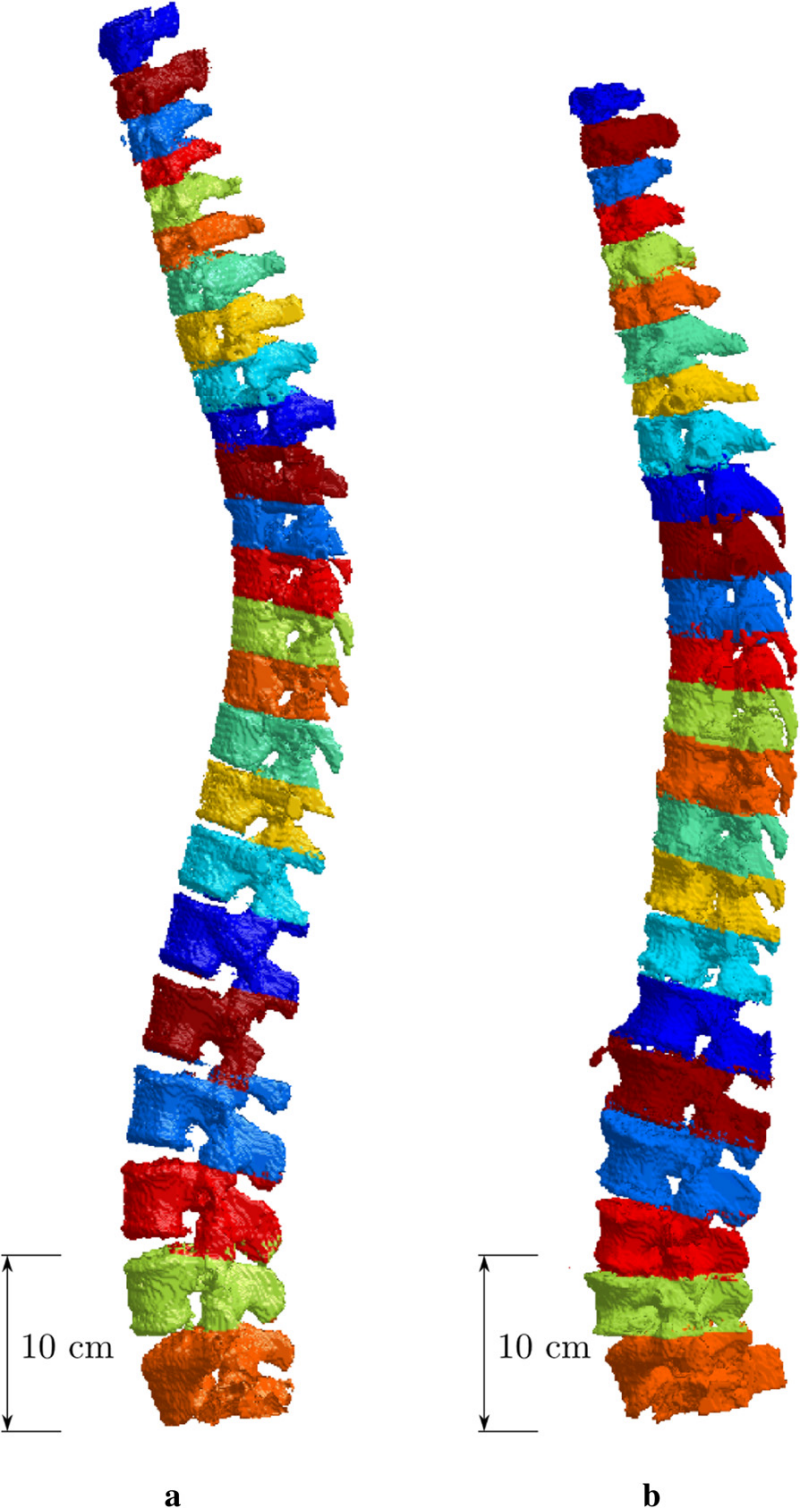
Integration examples on the two selected cases. The whole spines are segmented, with the 7 cervical, 12 thoracic, and 5 lumbar vertebrae. The volume rendering is interpolated from the binary segmentations and vertebral volumes are delimited with different colors, within the same color set. **a**: healthy spine. **b**: arthritic spine, with degenerative joint alterations, a thoracic Forestier’s disease, and a L4 compression

We present in this section examples of full-spine segmentations. Two CT scans acquired in clinical routine were selected: the first one does not present specificities in its spine and can be considered as a standard healthy case. The second one presents vertebral compression, which corresponds to a flattening of the inter-vertebral disk and occurs mostly with age. From each scan, we manually defined the vertebral bounding boxes so as to enclose the vertebral bodies. Each volume is processed separately and placed at its initial location. The final results are presented in Fig. 13.

First of all, one can notice on both results that the segmentations include some separation artifacts due to the delimitations of the volumes (initial bounding box). Thus, some vertebral elements have been segmented out from the total result, as they are initially badly delimited. Note also that some non-vertebral elements have been segmented in; this is in particular the case with the L1 vertebra from the second case which present calcifications (as in Fig. 12b). This also happens with surrounding bones, such as the ribs for thoracic vertebrae and the pelvis for the L5 vertebra. Nevertheless, the segmentation is not impacted by these inclusions and performs correctly.

From Fig. 13, the changes in vertebrae shape and size are clear within one patient and also between patients. Indeed, it is noticeable that the seven lower vertebrae shape differs between the two cases. The vertebral compression induces deformations in the observed vertebral bodies; thus, their shape does differ from prior expectations. Note also that the second case presents an arthrosis between T12 and L1, causing vertebral bodies to join. Despite these specificities, our method performs correctly on all vertebrae; all of the vertebrae sub-structure are clearly segmented.

The results show that the proposed method can be successfully integrated within a simple spine processing and thus can be used in more complex framework. Given the results from the previous sections, one can expect our method to perform well in most practical situations, regardless of the vertebra type, position, and specificity. Further discussion on the method is given in the next section.

## Discussion and conclusion

Vertebra segmentation is a challenging task. The wide range of shapes, the high rate of aging modifications, and the pathologic alterations frequently encountered in real cases explain the difficulties of an automatic segmentation in daily practice patients.

Hence, most of the published works about vertebra segmentation seem to be developed and evaluated on ideal data, namely in a young population in which vertebra are well separated, with CT providing a very high contrast between medullar bone, vertebra boundaries, and soft tissues. Additionally, sometimes only the lumbar spine is evaluated, with a consequent lack of information about the robustness of the presented schemes toward thoracic and cervical vertebrae.

This work is part of a larger project on spinal registration for patients presenting bone tumors. The segmentation method we present is thus developed in a real practice perspective, explaining why we took into account pathologic cases as well as the most frequent aging modifications, without any prior on vertebrae shape and luminance. Bounding box initialization method can be obtained automatically using state-of-the art techniques.

The presented method results fulfills the requirements of automatic bone segmentation prior to registration processes, with an affordable computational time.

## Endnotes

^1^Note that this operation is similar to a morphological gradient, with two different structuring elements instead of one.

^2^We use volume height since it is shorter than depth or width for the vertebral volumes we process.

^3^Experiments show that other perimeter-based path such as using a coronal axis slice by slice or using concentric helix leads to similar results.
